# Physico-chemical and rheological properties datasets related to batch mesophilic anaerobic digestion of waste activated sludge, primary sludge, and mixture of sludge with organic and inorganic matter

**DOI:** 10.1016/j.dib.2020.106418

**Published:** 2020-10-19

**Authors:** Samira Miryahyaei, Farihin Abdul Muthalib, Muhammad Syarifuddin Ab Aziz, Muhammad Shafeeq Ayub, Shirantha Jayaratna

**Affiliations:** Chemical and Environmental Engineering, School of Engineering, RMIT University, 3001 Melbourne, Australia

**Keywords:** Physico-chemical, Rheological properties, Mesophilic batch anaerobic digestion, Biochemical methane potential test

## Abstract

The datasets included in this paper provide periodically measured physico-chemical and rheological properties of mesophilic batch anaerobic digesters’ content for 30 days biochemical methane potential tests (BMP). Waste activated sludge (WAS) and primary sludge (PS) were the main substrates and digested sludge from a large scale mesophilic anaerobic digester was the inoculum. The substrates (F) and inoculum (I) were fed into the BMP rectors at different ratios of feed to inoculum (F/I = 1:1, 1:2, and 1:3). Experimental data on co-digestion of WAS with inorganic and organic additives were also reported. The reported characteristics such as total solids, volatile solids, total and soluble biochemical oxygen demand, ammonia, pH, as well as rheological properties over the duration of the BMP test could be used for analysing the changes in digestate properties as the anaerobic digestion process proceeds. The discussion and interpretation of the data have been provided in previous publications [[1], [2], [3]].

## Specifications Table

**Table:** 

Subject	Environmental Engineering
Specific subject area	Anaerobic Digestion, Biogas Production, Waste Management and Disposal, Biological Treatment, Bioenergy
Type of data	Tables
How data were acquired	The total and volatile solids (TS and VS) contents were measured according to the USEPA method 1684. The total and soluble chemical oxygen demand (tCOD and sCOD), volatile acids (VAs), and ammonia were analysed from colorimetric techniques using HACH reagents and DR5000 spectrophotometer following the HACH methods 8000, 8196, and 10031, respectively. The measurement of pH was conducted in a ThermoOrion (Model 550A) pH meter. The alkalinity was measured in accordance with the the APHA method 2320B. The volume of biogas was measured by water displacement method. To determine the biogas composition, biogas samples were analysed according to the APHA method 2720C by gas chromatography (GC-TID). The rheological measurements were conducted in TA Instruments DHR3 rheometer (stress-controlled). The zeta potential and bound water were measured by Zetasizer and Differential Scanning Calorimeter (DSC), respectively. A filtration rig was used to measure the Specific Resistance Filtration (SRF). A constant pressure on the samples was applied in the rig and the filtrate volume was recorded as a function of time.
Data format	Raw
Parameters for data collection	The data reported was collected during anaerobic digestion of the substrates WAS, PS and WAS+PS to occasionally evaluate the evolution of different properties and biogas yield over the process. All the digesters were operated under mesophilic conditions at 37±1˚C in shaker incubator at 100 rpm.
Description of data collection	Anaerobic digestion for each batch was conducted at the same temperature and mixing conditions. For each batch, 62-100 serum bottles were used to investigate the changes in properties and yield of biogas during the 30 days biochemical methane potential test (BMP). As degradation of readily degradable organic matter occurs fast, during the first two weeks, each batch was sampled twice a week during the first two weeks and once over the remaining weeks, the 3^rd^ and 4^th^ week.
Data source location	Institution: RMIT university City/Town/Region: Melbourne/Victoria Country: Australia
Data accessibility	The raw data deposited on Mendeley: https://data.mendeley.com/datasets/w5sn87zxmw/1
Related research article	Miryahyaei, S., Olinga, K., Abdul Muthalib, F. A., Das, T., Ab Aziz, M. S., Othman, M., Baudez, JC., Batstone, D. and Eshtiaghi (2019), N., Impact of rheological properties of substrate on anaerobic digestion and digestate dewaterability: New insights through rheological and physico-chemical interaction, Water Research, 10.1016/j.watres.2018.11.049[1] Miryahyaei, S., Olinga, K., Ayub, M. Sh., Jayaratna, Sh. Sh., Othman, M. and Eshtiaghi, N. (2020) Rheological measurements as indicator for hydrolysis rate, organic matter removal, and dewaterability of digestate in anaerobic digesters. Journal of Environmental Chemical Engineering, 103970 10.1016/j.jece.2020.103970[2] Miryahyaei, S., Das, T., Othman, M., Batstone, D. and Eshtiaghi, N. (2020) Anaerobic co-digestion of sewage sludge with cellulose, protein, and lipids: Role of rheology and digestibility. Journal of Science of Total Environment, 731, 139214, 10.1016/j.scitotenv.2020.139214[3]

## 1 Value of the Data

• These datasets include chemical and physical properties of digestate on different days of digestion, which should help other researchers to analyse/validate the potential relationship between the substrates and digestate properties and anaerobic digesters performance. For example, the dataset including chemical and physical properties of primary and waste activated sludge from two different plants, provides an insight into the impact of type and source of the substrate (i.e. sludge) on the different properties.
• The COD values, biogas production rate and biogas yield from the anaerobic digestion of the various substrates (PS, WAS, PS-WAS, cellulose, protein, lipids) could enable mass balance and performance assessment of the anaerobic digestion process to identify potential pathways for the organic matter valorization, specifically the PS and WAS without the need for carrying out the experimental work .
• The VS and TS data collected from different digesters on different digestion days for different types of sludge (PS and WAS) and from different sources could help to explore the relationship between degradation and other physico-chemical properties such as ammonia, pH, tCOD, sCOD, flow behavior, and viscoelasticity. It could also assist to develop an understanding of characterization of degradation based on COD measurement and VS and which one is a better indicator of the degradability. In addition, this data can be used to establish correlations for biogas production, VS measurement and degradability, which can help to inform the design of experiments involving similar substrates and to predict biogas production at similar conditions. Overall, these datasets can be used to carry out preliminary assessment of performance of digesters at wastewater treatment plants and validate the analysis of data reported in other related studies on the performance of these digesters.
• These datasets can be used by both researchers and designers of anaerobic digesters because it helps to understand the effects of the addition of different organic wastes (cellulose, protein, and lipids) and an inorganic additive (acrylic powder) on digestion of sludge to develop more innovative approach for enriching anaerobic digestion process.
• These datasets include rheological properties of sludge over the digestion time, which helps to design a modulated mixing for anaerobic digesters and optimise pump performance via utilising CFD simulation.

## 2 Data Description

The datasets are categorised into two anaerobic digestion processes: mono-digestion (Supplementary File 1A and 2A) and co-digestion (Supplementary File 1B and 2B).

The mono-digestion process is related to digestion of waste activated sludge (WAS) and primary sludge (PS) as the main substrates which were collected from two wastewater treatment plants - the Eastern Treatment Plant (ETP) and Mount Martha (MM) Treatment Plant, Melbourne, Australia. The ratio of feed to inoculum (F/I) varied between the digesters. For each substrate, three digesters of different F/I ratios (1:1, 1:2, 1:3, VS basis) were carried out. The average values for the physico-chemical and rheological properties of feed and digestate for all digesters are summarised in Supplementary File 1A, which includes Tables from 1A.1 to 1A.14, titled as follows:

Table 1A.1. Physiochemical characteristics of primary sludge, waste activated sludge collected from ETP and MM; and physiochemical characteristics of digested sludge (inoculum) collected from ETP

Table 1A.2. Change of Total Solids (TS, %) of digester content for 30 days BMP test of two different types of sludge (Primary sludge and Waste activated sludge) as substrate at different F/I ratios (1:1, 1:2, 1:3)

Table 1A.3. Change of Volatile Solids (VS/TS %) of digester content for 30 days BMP test of two different types of sludge (Primary sludge and Waste activated sludge) as substrate at different F/I ratios (1:1, 1:2, 1:3)

Table 1A.4. Change of Soluble Organic Matter (SCOD, mg O_2_/L) for 30 days BMP test of two different types of sludge (Primary sludge and Waste activated sludge) as substrate at different F/I ratios (1:1, 1:2, 1:3)

Table 1A.5. Change of Total Organic Matter (TCOD, mg O_2_/L) for 30 days BMP test of two different types of sludge (Primary sludge and Waste activated sludge) as substrate at different F/I ratios (1:1, 1:2, 1:3)

Table 1A.6. Change of pH (-) for 30 days BMP test of two different types of sludge (Primary sludge and Waste activated sludge) as substrate at different F/I ratios (1:1, 1:2, 1:3)

Table 1A.7. Change of Ammonia (mg/L) for 30 days BMP test of two different types of sludge (Primary sludge and Waste activated sludge) as substrate at different F/I ratios (1:1, 1:2, 1:3)

Table 1A.8. Zeta Potential (mV) at the start day of BMP test of two different types of sludge (Primary sludge and Waste activated sludge) as substrate at different F/I ratios (1:1, 1:2, 1:3)

Table 1A.9. Biogas yield (mL/g VS) for 30 days BMP test of two different types of sludge (Primary sludge and Waste activated sludge) as substrate at different F/I ratios (1:1, 1:2, 1:3)

Table 1A.10. Change of yield stress (Pa) of digester content for 30 days BMP test of two different types of sludge (Primary sludge and Waste activated sludge) as substrate at different F/I ratios (1:1, 1:2, 1:3)

Table 1A.11. Stress Sweep Test - DAY 0, Sweep data of Torque (µN.m), and angular velocity (rad/s), shear stress (Pa) and shear rate (s^−1^) of digester content at the start day of BMP test of two different types of sludge (Primary sludge and Waste activated sludge) as substrate at different F/I ratios (1:1, 1:2, 1:3)

Table 1A.12. Stress Sweep Test - DAY 3, Sweep data of Torque (µN.m), and angular velocity (rad/s), shear stress (Pa) and shear rate (s^−1^) of digester content on Day 3 of BMP test of two different types of sludge (Primary sludge and Waste activated sludge) as substrate at different F/I ratios (1:1, 1:2, 1:3)

Table 1A.13. Stress Sweep Test - DAY 17, Sweep data of Torque (µN.m), and angular velocity (rad/s), shear stress (Pa) and shear rate (s^−1^) of digester content on Day 17 of BMP test of two different types of sludge (Primary sludge and Waste activated sludge) as substrate at different F/I ratios (1:1, 1:2, 1:3)

Table 1A.14. Stress Sweep Test-DAY 30, Sweep data of Torque (µN.m), and angular velocity (rad/s), shear stress (Pa) and shear rate (s^−1^) of digester content on Day 30 of BMP test of two different types of sludge (Primary sludge and Waste activated sludge) as substrate at different F/I ratios (1:1, 1:2, 1:3)

There are also four digesters of WAS with the same F/I ratio (1:1) and different total solid content (2.1, 3.4, 4.1, 5.02%), for which data are reported in Supplementary File 2A with the following list of included tables (Table 2A.1 - 2A.21).

Table 2A.1. Physiochemical characteristics of waste activated sludge and digested sludge (inoculum) collected from ETP

Table 2A.2. Change of Total Solids (TS %) of digester content for 30 days BMP test of Waste activated sludge (at different total solid content 2.1, 3.4, 4.1, 5.02 wt% TS) as substrate at F/I ratio of 1:1

Table 2A.3. Change of Volatile Solids (VS/TS %) of digester content for 30 days BMP test of Waste activated sludge (at different total solid content 2.1, 3.4, 4.1, 5.02 wt% TS) as substrate at F/I ratio of 1:1

Table 2A.4. Change of Soluble Organic Matter (SCOD, mg O_2_/L) for 30 days BMP test of Waste activated sludge (at different total solid content 2.1, 3.4, 4.1, 5.02 wt% TS) as substrate at F/I ratio of 1:1

Table 2A.5. Change of Total Organic Matter (TCOD, mg O_2_/L) for 30 days BMP test of Waste activated sludge (at different total solid content 2.1, 3.4, 4.1, 5.02 wt% TS) as substrate at F/I ratio of 1:1

Table 2A.6. Change of pH (-) for 30 days BMP test of Waste activated sludge (at different total solid content 2.1, 3.4, 4.1, 5.02 wt% TS) as substrate at F/I ratio of 1:1

Table 2A.7. Change of Ammonia (mg/L) for 30 days BMP test of Waste activated sludge (at different total solid content 2.1, 3.4, 4.1, 5.02 wt% TS) as substrate at F/I ratio of 1:1

Table 2A.8. Change of Zeta Potential (mV) for 30 days BMP test of Waste activated sludge (at different total solid content 2.1, 3.4, 4.1, 5.02 wt% TS) as substrate at F/I ratio of 1:1

Table 2A.9. Change of Bound water (g/g) for 30 days BMP test of Waste activated sludge (at different total solid content 2.1, 3.4, 4.1, 5.02 wt% TS) as substrate at F/I ratio of 1:1

Table 2A.10. The Specific Filtration Resistance (SRF, mL/g) of digested sludge after 30days BMP test of Waste activated sludge (at different total solid content 2.1, 3.4, 4.1, 5.02 wt% TS) as substrate at F/I ratio of 1:1

Table 2A.11. Biogas yield (mL/g VS) for 30 days BMP test of Waste activated sludge (at different total solid content 2.1, 3.4, 4.1, 5.02 wt% TS) as substrate at F/I ratio of 1:1

Table 2A.12. Change of yield stress (Pa), flow consistency index K (Pa.s^n^) and flow behaviour index n (-) of digester content for 30 days BMP test of Waste activated sludge (at different total solid content 2.1, 3.4, 4.1, 5.02 wt% TS) as substrate at F/I ratio of 1:1

Table 2A.13. Change of viscoelastic properties (G’ and G”, Pa) of digester content for 30 days BMP test of Waste activated sludge (at different total solid content 2.1, 3.4, 4.1, 5.02 wt% TS) as substrate at F/I ratio of 1:1

Table 2A.14. Stress Sweep Test - DAY 0, Sweep data of Torque (µN.m), and angular velocity (rad/s), shear stress (Pa) and shear rate (s^−1^) of digester content at the start day of BMP test of Waste activated sludge (at different total solid content 2.1, 3.4, 4.1, 5.02 wt% TS) as substrate at F/I ratio of 1:1

Table 2A.15. Stress Sweep Test - DAY 3, Sweep data of Torque (µN.m), and angular velocity (rad/s), shear stress (Pa) and shear rate (s^−1^) of digester content on Day 3 of BMP test of Waste activated sludge (at different total solid content 2.1, 3.4, 4.1, 5.02 wt% TS) as substrate at F/I ratio of 1:1

Table 2A.16. Stress Sweep Test - DAY 17, Sweep data of Torque (µN.m), and angular velocity (rad/s), shear stress (Pa) and shear rate (s^−1^) of digester content on Day 17 of BMP test of Waste activated sludge (at different total solid content 2.1, 3.4, 4.1, 5.02 wt% TS) as substrate at F/I ratio of 1:1

Table 2A.17. Stress Sweep Test-DAY 30, Sweep data of Torque (µN.m), and angular velocity (rad/s), shear stress (Pa) and shear rate (s^−1^) of digester content on Day 30 of BMP test of Waste activated sludge (at different total solid content 2.1, 3.4, 4.1, 5.02 wt% TS) as substrate at F/I ratio of 1:1

Table 2A. 18. Amplitude Sweep Test - DAY 0, (G’(Pa), G” (Pa) and strain (%)) of digester content at the start day of BMP test of Waste activated sludge (at different total solid content 2.1, 3.4, 4.1, 5.02 wt% TS) as substrate at F/I ratio of 1:1

Table 2A. 19. Amplitude Sweep Test - DAY 3, (G’(Pa), G” (Pa) and strain (%)) of digester content on Day 3 of BMP test of Waste activated sludge (at different total solid content 2.1, 3.4, 4.1, 5.02 wt% TS) as substrate at F/I ratio of 1:1

Table 2A. 20. Amplitude Sweep Test - DAY 17, (G’(Pa), G” (Pa) and strain (%)) of digester content on Day 17 of BMP test of Waste activated sludge (at different total solid content 2.1, 3.4, 4.1, 5.02 wt% TS) as substrate at F/I ratio of 1:1

Table 2A. 21. Amplitude Sweep Test - DAY 30, (G’(Pa), G” (Pa) and strain (%)) of digester content on Day 30 of BMP test of Waste activated sludge (at different total solid content 2.1, 3.4, 4.1, 5.02 wt% TS) as substrate at F/I ratio of 1:1

The co-digestion process reports the co-digestion of WAS with different amounts of inorganic matter (Poly Methyl Methacrylate powder, supplied by Sigma Aldrich) as well as co-digestion of PS and WAS (mixed based on a 1:1 gVS:gVS ratio) with organic matter of different digestibility i.e., cellulose, protein, or lipids. Alpha-cellulose powder (supplied by Sigma Aldrich), gelatine powder (supplied by ThermoFisher Scientific Australia), and oleic acid (supplied by Sigma Aldrich) were used as the respective cellulose, protein and lipid additives mixed with WAS. The performance of five digesters fed by different ratios of inorganic matter: WAS are compared and summarized in Supplementary File 1B; and the performance of 15 digesters that received different ratios of organic matter: PS-WAS are listed in Supplementary File 2B. For these anaerobic co-digestion tests, the biogas was regularly analysed, while the properties of the digestate were tested at the end of the experiment. In all experiments, the digested sludge (DS) used as inoculum was collected from the Eastern Treatment Plant. Duplicates were carried out for each condition tested. All the tests were conducted in duplicate on the day of sampling to eliminate storage impact. All the supplementary files include the average values for the physico-chemical and rheological properties. Supplementary File 1B includes Tables from 1B.1 to 1B.24, and Supplementary File 2B includes Tables from 2B.1 to 2B.15 titled as follows:

Table 1B.1. Physiochemical characteristics of waste activated sludge and digested sludge (inoculum) collected from ETP in autumn season (March)

Table 1B.2. Change of Total Solids (TS %) of digester content for 30 days BMP test of Waste activated sludge with different inorganic loading of 0.03, 0.07,0.11 and 0.2 gPMMA/gWAS as substrate at F/I ratio of 1:1.

Table 1B.3. Change of Volatile Solids (VS/TS %) of digester content for 30 days BMP test of Waste activated sludge with different inorganic loading of 0.03, 0.07,0.11 and 0.2 gPMMA/gWAS as substrate at F/I ratio of 1:1.

Table 1B.4. Change of Soluble Organic Matter (SCOD, mg O_2_/L) of digester content for 30 days BMP test of Waste activated sludge with different inorganic loading of 0.03, 0.07,0.11 and 0.2 g PMMA/g WAS as substrate at F/I ratio of 1:1.

Table 1B.5. Change of Total Organic Matter (TCOD, mg O_2_/L) of digester content for 30 days BMP test of Waste activated sludge with different inorganic loading of 0.03, 0.07,0.11 and 0.2 g PMMA/g WAS as substrate at F/I ratio of 1:1.

Table 1B.6. Change of pH (-) of digester content for 30 days BMP test of Waste activated sludge with different inorganic loading of 0.03, 0.07,0.11 and 0.2 gPMMA/gWAS as substrate at F/I ratio of 1:1.

Table 1B.7. Change of Ammonia (mg/L) for 30 days BMP test of Waste activated sludge with different inorganic loading of 0.03, 0.07,0.11 and 0.2 gPMMA/gWAS as substrate at F/I ratio of 1:1.

Table 1B.8. Change of Zeta Potential (mV) of digester content for 30 days BMP test of Waste activated sludge with different inorganic loading of 0.03, 0.07,0.11 and 0.2 gPMMA/gWAS as substrate at F/I ratio of 1:1.

Table 1B.9. Change of Bound water (g/g) of digester content for 30 days BMP test of Waste activated sludge with different inorganic loading of 0.03, 0.07,0.11 and 0.2 gPMMA/gWAS as substrate at F/I ratio of 1:1.

Table 1B.10. The Specific volume Index (SVI, mL/g) of digested sludge after 30days BMP test of Waste activated sludge with different inorganic loading of 0.03, 0.07,0.11 and 0.2 gPMMA/g WAS as substrate at F/I ratio of 1:1.

Table 1B.11. The water content (%) of digested sludge after 30days BMP test of Waste activated sludge with different inorganic loading of 0.03, 0.07,0.11 and 0.2 gPMMA/gWAS as substrate at F/I ratio of 1:1.

Table 1B.12. Biogas yield (mL/g VS) for 30 days BMP test of Waste activated sludge with different inorganic loading of 0.03, 0.07,0.11 and 0.2 gPMMA/gWAS as substrate at F/I ratio of 1:1.

Table 1B.13. Change of yield stress (Pa), flow consistency index K (Pa.s^n^) and flow behaviour index n (-) of digester content for 30 days BMP test of Waste activated sludge with different inorganic loading of 0.03, 0.07,0.11 and 0.2 gPMMA/gWAS as substrate at F/I ratio of 1:1.

Table 1B.14. Change of viscoelastic properties (G’ and G”, Pa) of digester content for 30 days BMP test of Waste activated sludge with different inorganic loading of 0.03, 0.07,0.11 and 0.2 gPMMA/gWAS as substrate at F/I ratio of 1:1.

Table 1B.15. Stress Sweep Test - DAY 0, Sweep data of Torque (µN.m), and angular velocity (rad/s), shear stress (Pa) and shear rate (s^−1^) of digester content at the start day of BMP test of Waste activated sludge with different inorganic loading of 0.03, 0.07,0.11 and 0.2 gPMMA/gWAS as substrate at F/I ratio of 1:1.

Table 1B.16. Stress Sweep Test - DAY 2, Sweep data of Torque (µN.m), and angular velocity (rad/s), shear stress (Pa) and shear rate (s^−1^) of digester content on Day 2 of BMP test of Waste activated sludge with different inorganic loading of 0.03, 0.07,0.11 and 0.2 gPMMA/gWAS as substrate at F/I ratio of 1:1.

Table 1B.17. Stress Sweep Test - DAY 7, Sweep data of Torque (µN.m), and angular velocity (rad/s), shear stress (Pa) and shear rate (s^−1^) of digester content on Day 7 of BMP test of Waste activated sludge with different inorganic loading of 0.03, 0.07,0.11 and 0.2 gPMMA/gWAS as substrate at F/I ratio of 1:1.

Table 1B.18. Stress Sweep Test - DAY 17, Sweep data of Torque (µN.m), and angular velocity (rad/s), shear stress (Pa) and shear rate (s^−1^) of digester content on Day 17 of BMP test of Waste activated sludge with different inorganic loading of 0.03, 0.07,0.11 and 0.2 gPMMA/gWAS as substrate at F/I ratio of 1:1.

Table 1B.19. Stress Sweep Test-DAY 30, Sweep data of Torque (µN.m), and angular velocity (rad/s), shear stress (Pa) and shear rate (s^−1^) of digester content on Day 30 of BMP test of Waste activated sludge with different inorganic loading of 0.03, 0.07,0.11 and 0.2 gPMMA/gWAS as substrate at F/I ratio of 1:1.

Table 1B. 20. Amplitude Sweep Test - DAY 0, (G’(Pa), G” (Pa) and strain (%)) of digester content at the start day of BMP test of Waste activated sludge with different inorganic loading of 0.03, 0.07,0.11 and 0.2 gPMMA/gWAS as substrate at F/I ratio of 1:1.

Table 1B. 21. Amplitude Sweep Test - DAY 2, (G’(Pa), G” (Pa) and strain (%)) of digester content on Day 2 of BMP test of Waste activated sludge with different inorganic loading of 0.03, 0.07,0.11 and 0.2 gPMMA/gWAS as substrate at F/I ratio of 1:1

Table 1B. 22. Amplitude Sweep Test - DAY 3, (G’(Pa), G” (Pa) and strain (%)) of digester content on Day 3 of BMP test of Waste activated sludge with different inorganic loading of 0.03, 0.07,0.11 and 0.2 gPMMA/gWAS as substrate at F/I ratio of 1:1

Table 1B. 23. Amplitude Sweep Test - DAY 17, (G’(Pa), G” (Pa) and strain (%)) of digester content on Day 17 of BMP test of Waste activated sludge with different inorganic loading of 0.03, 0.07,0.11 and 0.2 gPMMA/gWAS as substrate at F/I ratio of 1:1

Table 1B. 24. Amplitude Sweep Test - DAY 30, (G’(Pa), G” (Pa) and strain (%)) of digester content on Day 30 of BMP test of Waste activated sludge with different inorganic loading of 0.03, 0.07,0.11 and 0.2 gPMMA/gWAS as substrate at F/I ratio of 1:1

Supplementary File 2B includes Tables from 2B.1 to 2B.15 as of the following:

Table 2B.1. Physiochemical characteristics of waste activated sludge, primary sludge and digested sludge (inoculum) collected from ETP

Table 2B.2. Change of Total Solids (TS %) of digester content after 30 days BMP test of co-digestion of primary sludge and waste activated sludge (PS-WAS) at different co-substrate (cellulose, protein, and lipids) to mixed sludge ratios of 2% to 8 wt% compared to the start day

Table 2B.3. Change of Volatile Solids (VS/TS %) of digester content after 30 days BMP test of co-digestion of primary sludge and waste activated sludge (PS-WAS) at different co-substrate (cellulose, protein, and lipids) to mixed sludge ratios of 2% to 8 wt% compared to the start day

Table 2B.4. Soluble Organic Matter (sCOD, mg O_2_/L) of digester content at the start of BMP test of co-digestion of primary sludge and waste activated sludge (PS-WAS) at different co-substrate (cellulose, protein, and lipids) to mixed sludge ratios of 2% to 8 wt% compared to the start day

Table 2B.5. Total Organic Matter (tCOD, mg O_2_/L) of digester content at the start of BMP test of co-digestion of primary sludge and waste activated sludge (PS-WAS) at different co-substrate (cellulose, protein, and lipids) to mixed sludge ratios of 2% to 8 wt% compared to the start day

Table 2B.6. pH (-) of of digester content at the start of BMP test of co-digestion of primary sludge and waste activated sludge (PS-WAS) at different co-substrate (cellulose, protein, and lipids) to mixed sludge ratios of 2% to 8 wt% compared to the start day

Table 2B.7. Ammonia (mg/L) of digester content at the start of BMP test of co-digestion of primary sludge and waste activated sludge (PS-WAS) at different co-substrate (cellulose, protein, and lipids) to mixed sludge ratios of 2% to 8 wt% compared to the start day

Table 2B.8. Volatile acid (mg acetic acid/L) of digester content after 30 days BMP test of co-digestion of primary sludge and waste activated sludge (PS-WAS) at different co-substrate (cellulose, protein, and lipids) to mixed sludge ratios of 2% to 8 wt%

Table 2B.9. Alkalinity (mg CaCO_3_/L) of digester content after 30 days BMP test of co-digestion of primary sludge and waste activated sludge (PS-WAS) at different co-substrate (cellulose, protein, and lipids) to mixed sludge ratios of 2% to 8 wt%

Table 2B.10. The VA/alkalinity (-) of digester content after 30 days BMP test of co-digestion of primary sludge and waste activated sludge (PS-WAS) at different co-substrate (cellulose, protein, and lipids) to mixed sludge ratios of 2% to 8 wt%

Table 2B.11. Biogas yield (mL/g VS) for 30 days BMP test of co-digestion of primary sludge and waste activated sludge (PS-WAS) at different co-substrate (cellulose, protein, and lipids) to mixed sludge ratios of 2% to 8 wt%

Table 2B.12. Methane content of biogas (%) Day 30 day of BMP test of co-digestion of primary sludge and waste activated sludge (PS-WAS) at different co-substrate (cellulose, protein, and lipids) to mixed sludge ratios of 2% to 8 wt%

Table 2B.13. Yield stress (Pa), flow consistency index K (Pa.s^n^) and flow behaviour index n (-) of digester content on Day 30 of BMP test of co-digestion of primary sludge and waste activated sludge (PS-WAS) at different co-substrate (cellulose, protein, and lipids) to mixed sludge ratios of 2% to 8 wt% compared to the start day

Table 2B.14. Stress Sweep Test - DAY 0, Sweep data of Torque (µN.m), and angular velocity (rad/s), shear stress (Pa) and shear rate (s^−1^) of digester content on Day 0 of BMP test of co-digestion of primary sludge and waste activated sludge (PS-WAS) at different co-substrate (cellulose, protein, and lipids) to mixed sludge ratios of 2% to 8 wt%

Table 2B.15. Stress Sweep Test - DAY 30, Sweep data of Torque (µN.m), and angular velocity (rad/s), shear stress (Pa) and shear rate (s^−1^) of of digester content on Day 0 of BMP test of co-digestion of primary sludge and waste activated sludge (PS-WAS) at different co-substrate (cellulose, protein, and lipids) to mixed sludge ratios of 2% to 8 wt%

## 3 Experimental Design, Materials, and Methods

### 3.1 Experimental Design and set up

The batch digestion experiments in this study were carried out under anaerobic conditions using 250 mL serum bottles. For each batch, 62-100 serum bottles (digesters) were set-up to allow for analysis of certain parameters at regular intervals and to monitor the evolution of different properties as digestion proceeded. Two digesters containing only inoculum were included in the BMP test to account for the methane potential of the added inoculum. All digesters were flushed with nitrogen [4] and sealed with rubber Suba-Seal, 16 mm, Sigma-Aldrich, before incubating in a shaker incubator at 100 rpm, 37 ± 1^∘^C, for 30 days of biomethane potential (BMP) test.

### 3.2 Analytical Procedure of Measuring Physico-Chemical Properties

The daily biogas production was measured using a water displacement unit. Gas chromatograph (Varian 450-GC, Varian Australia Pty Ltd., the Netherlands) with a column (GS Carbonplot 113–3132, 1.5 mm, 30 m × 0.320 mm, stainless steel, Agilent Technologies Inc., Australia) was used to measure the compositions of biogas samples according to the APHA method 2720C. The carrier gas was Helium with a flow rate of 28 mL/min. The temperatures in the column, detector, and injector were maintained at 70 °C, 200 °C and 100 °C, respectively.

Total solids (TS) and volatile solids (VS) were measured according to USEPA method 1684 (EPA 2010). The sludge samples were dried in an oven at 105°C for 24 h for measurement of TS. To measure VS, the residues were burned in a furnace at 550°C for 2 h.

The samples were diluted 40 times with deionized water to measure the total chemical oxygen demand (tCOD). For the analysis of soluble chemical oxygen demand (sCOD), the samples were filtered using a syringe filter (0.45 μm). Both tCOD and sCOD measurements were performed according to HACH procedures (COD high range plus reagents, DR5000 Spectrophotometer and DRB200 digester).

The pH was measured in a calibrated pH meter (ThermoOrion, Model 550A).

For the measurement of ammonia, the samples were centrifuged at 10,000 rpm (13,000 g) for 5 min followed by filtration using a syringe filter (0.45 μm). The filtrate was the diluted 40 times with deionized water. The ammonia content was analysed according to the HACH method 10031 using a HACH spectrophotometer (Model DR/4000 U).

The alkalinity was measured according to APHA method 2320B. 20mL of the filtrate was titrated from pH 7−8.3 to pH 4.5 with H_2_SO_4_.The alkalinity was then calculated using Eq. (1) [5]:

(1) $$\begin{array}{ccc} Alkalinity\;\left(mg\;CaCO_{3}/L\right) & = & \;Amount\;of\;acid\;used\;to\;reach\;pH\;4.5\;\left(ml\right)\;\times Normality\;of\;acid\;\left(eq/L\right)\;\\ & & \times \;50,000\;\left(mg\;CaCO_{3}/eq\right)\;/\;sample\;volume\;\left(ml\right) \end{array}$$

The zeta potential of the samples were measured using Zetasizer, Malvern instrument. The samples was centrifuged at 10000 rpm (13000 g) for 5 min before transferring to the disposable folded capillary cells (DTS1070) to avoid settlement of particulate matter on the measurement cell [6].

The bound water of the samples were measured using a Differential Scanning Calorimeter (DSC), TA instruments. 20 mg sludge sample was cooled from 20°C to -20°C at a cooling ramp of -5°C/min [7] and then raised up to 20°C at the same temperature ramp. The released heat during freezing of water was recorded. The bound water was obtained by Eq. (2).

(2) $$W_{B}=W_{T}-\Delta H/\Delta H_{o}$$

where, *W_T_* (g/g dry sludge) is the quotient of total water and dry sludge (105°C overnight), *ΔH* (J/g) is the exothermic area and *ΔH_o_* (334.7 J/g) is the standard fusion heat of water [8].

The settleability was determined from the Sludge Volume Index (SVI). The Sludge Volume Index (*SVI*) can be calculated using Eq. (3) [9] from the height of the settled sludge after 30 min of settling.

(3) $$SVI=\left(100\;\times H_{30}\right)/\left(H_{0}\times X_{0}\right)$$

where, *H_30_* (cm) is the height after 30 min of settling, *H_0_* (cm) is the initial height and *X_0_* (%) is the solids concentration of the sample.

Centrifugal dewatering method [10] was used to measure the water content of the samples. 40 mL sample was centrifuged at 1500 g for 10 min [10], and then dried at 105°C overnight. Water content of dewatered sludge cake can be calculated from Eq. (4) [9]:

(4) $$WC\%=\left(1-\frac{M_{dried\;sludge}}{M_{sludge\;cake}}\right)\times \;100\%$$

where, *M_dried sludge_* is the weight of the dried sludge and *M_sludge cake_* is the weight of the sludge cake obtained by centrifugation, in grams.

The Specific Filtration Resistance (*SRF*) was measured using a filtration rig. A constant pressure of 130 kPa was applied to the sample by a pneumatically driven piston, forcing the filtrate to pass through a Millipore membrane (0.45 µm pore size, and 47 mm diameter). The volume of the filtrate released by the applied pressure was automatically recorded over the time of filtration. The *SRF* was then obtained by Eq. (5) [11].

(5) $$SRF=2PA^{2}b\;/\;\mu \omega$$

Where, *P* (Pa) is the pressure, *A* (m^2^) is the area of membrane, *μ* (kg.s/m^2^) is the viscosity of the filtrate assumed as the viscosity of water, *ω* (kg/m^3^) is the concentration of cake on the filtrate per unit volume sludge, and *b* is calculated from the slope of the linear region of the graph of filtration time against the square of volume of filtrate.

### 3.3 Analytical Procedure of Measuring Rheological Properties

The rheological parameters were measured by a stress-controlled DHR3 rheometer (TA Instruments). The sample temperature during measurements were maintained using a water bath. A wide gap geometry with cup and grooved bob (bob diameter: 15 mm, cup diameter: 30 mm, bob length: 44 mm) was chosen to reduce the interference of particles content of sample suspension in the data. All the rheological tests were conducted following the same procedure: the samples (30 mL) were pre-sheared for 150 s at 300 s^−1^ followed by 5 min equilibrium at zero shear rate [12] to erase material memory and ensure reproducible data. For the analysis of viscoelasticity, the amplitude sweep oscillation tests were carried out in which a strain ramp from 1 to 300% under constant frequency of 1 Hz was used with 30 points per decade data collection. Then the samples were pre-sheared for 150s at 300 s^−1^ followed by 1 min equilibrium at zero shear rate, and the stress sweep test was performed to determine the flow behavior. A decreasing logarithmic ramp of shear rate from 300 s^−1^ to 1 s^−1^ was applied with 10s of measurement duration for each point. The temperature was kept at 25±1°C during all the rheological tests.

In the spreadsheets attached to this paper, the raw data from stress sweep test (shear stress, shear rate, torque, and angular velocity) are reported, which were obtained from the rheometer. For a gap in which *R_c_ − R_b_ ≤ R_b_*, the shear stress and shear rate needs to be recalculated. The shear stress (Pa) applied on the bob can be recalculated from the torque values and bob geometry using Eq. (6) [13]:

(6) $$\tau_{b}\left(M\right)=\frac{M}{2\pi hR_{b}^{2}}$$

where, M is the torque (N.m), h is the length of bob (m) and R_b_ is the radius of bob (m).

The shear rate (s^−1^) at the bob surface can also be recalculated using Krieger's Method [13]:

(7) $${\dot{\gamma }}_{b}=\frac{2\Omega }{n}\frac{\alpha^{2\;/\;n}}{\alpha^{2\;/\;n}-1}$$

where, Ω is the angular velocity (rad/s), α is the ratio of cup to bob radius, and n is the derivative of ln *τ_b_* with respect to *ln Ω*.

The flow properties (yield stress, flow consistency index, and flow behavior index) reported in the published linked paper have been calculated from the corrected shear stress and shear rate values obtained from Eqs. (6) and (7). However, the shear stress and shear rate data that is extracted from the rheometer are also added to all the tables just for an indication of values. Herschel–Bulkley model (Eq. 8) was used for developing flow behavior of samples:

(8) $$\tau =\tau_{y}+\;k{\dot{\gamma }}^{n}$$

where, *τ_y_* is the yield stress (Pa), *k* is the consistency index (Pa∙ s^n^), and *n* (-) is the flow behavior index.

## CRediT authorship contribution statement

**Samira Miryahyaei:** Methodology, Formal analysis, Investigation, Writing – review & editing. **Farihin Abdul Muthalib:** Investigation. **Muhammad Syarifuddin Ab Aziz:** Investigation. **Muhammad Shafeeq Ayub:** Investigation. **Shirantha Jayaratna:** Investigation.

## Declaration of Competing Interest

The authors declare that they have no competing interests.

## Appendix Supplementary materials

Image, application 1

Image, application 2

Image, application 3

Image, application 4
